# Case report: A family of atypical hemolytic uremic syndrome involving a *CFH::CFHR1* fusion gene and *CFHR3-1-4-2* gene duplication

**DOI:** 10.3389/fimmu.2024.1360855

**Published:** 2024-03-08

**Authors:** Yuko Tasaki, Hiroshi Tsujimoto, Tadafumi Yokoyama, Naotoshi Sugimoto, Shinji Kitajima, Hiroshi Fujii, Yoshihiko Hidaka, Noritoshi Kato, Shoichi Maruyama, Norimitsu Inoue, Taizo Wada

**Affiliations:** ^1^Department of Pediatrics, School of Medicine, Institute of Medical, Pharmaceutical, and Health Sciences, Kanazawa University, Kanazawa, Japan; ^2^Department of Molecular Genetics, Wakayama Medical University, Wakayama, Japan; ^3^Department of Nephrology and Rheumatology, Kanazawa University, Kanazawa, Japan; ^4^Department of Nephrology and Rheumatology, Ishikawa Prefectural Central Hospital, Kanazawa, Japan; ^5^Department of Nephrology, Nagoya University Graduate School of Medicine, Nagoya, Japan

**Keywords:** atypical hemolytic uremic syndrome, factor H, complement factor H-related, multiplex ligation-dependent probe amplification (MLPA), *CFH::CFHR1* fusion gene, *CFHR3-1-4-2* genes duplication

## Abstract

Mutations in the complement factor H (*CFH*) gene are associated with complement dysregulation and the development of atypical hemolytic uremic syndrome (aHUS). Several fusion genes that result from genomic structural variation in the *CFH* and complement factor H-related (*CFHR*) gene regions have been identified in aHUS. However, one allele has both *CFHR* gene duplication and *CFH::CFHR1* fusion gene have not been reported. An 8-month-old girl (proband) presented with aHUS and was treated with ravulizumab. Her paternal grandfather developed aHUS previously and her paternal great grandmother presented with anti-neutrophil cytoplasmic antibody-associated vasculitis and thrombotic microangiopathy (TMA). However, the proband’s parents have no history of TMA. A genetic analysis revealed the presence of *CFH::CFHR1* fusion gene and a *CFHR3-1-4-2* gene duplication in the patient, her father, and her paternal grandfather. Although several fusion genes resulting from structural variations of the *CFH–CFHR* genes region have been identified, this is the first report of the combination of a *CFH::CFHR1* fusion gene with *CFHR* gene duplication. Because the *CFH–CFHR* region is highly homologous, we hypothesized that *CFHR* gene duplication occurred. These findings indicate a novel pathogenic genomic structural variation associated with the development of aHUS.

## 1 Introduction

Atypical hemolytic uremic syndrome (aHUS) is a form of thrombotic microangiopathy (TMA) that results from the defective regulation of the alternative complement pathway. aHUS is characterized by microangiopathic hemolytic anemia, thrombocytopenia, and renal dysfunction (1–3). Genetic abnormalities associated with complement activation in the alternative pathway occur in 50%–60% of aHUS patients (1).

The complement factor H (FH) has an important role in regulating the alternative pathway and is considered a common genetic factor underlying aHUS (3, 4). With respect to cases of aHUS with complement factor H (*CFH*) gene mutations, prognosis is poor and affected patients progress to end-stage renal disease if not treated appropriately (5).

The *CFH* gene along with genes for complement Factor H-related (FHR) proteins 1, 2, 3, 4, and 5, are located within the regulators of complement activation (RCA) cluster on chromosome 1q32 (3, 6). Sequence analyses have revealed numerous duplications in this region because of the high degree of sequence homology. Nonallelic homologous recombination (NAHR) events occur within this repetitive sequence, which result in the formation of a *CFH::CFHR* fusion gene. The primary mutations in the *CFH* gene typically occur within the final one to three short consensus repeats (SCRs) (3, 7). Several fusion genes resulting from NAHR in the *CFH–CFHR* gene region have been identified, including *CFH::CFHR1*, *CFH::CFHR3*, and *CFHR1::CFH* (3, 8–12).

There is some confusion in the nomenclature of the *CFH* gene. The very small exon (Exon 10) within CFH gene only codes for 4 amino acids, forming the splicing isoform of FHL-1 (13). In this report, we designated the number of exons of the *CFH* gene as 23 including this very small exon. Three types of *CFH::CFHR1* fusion genes have been reported, initially described by *Venales et al.* (3, 8), where the two last exons of *CFH* are replaced with exons 5-6 of *CFHR1*. The second *CFH::CFHR1* fusion gene, reported by *Maga et al.* (3, 9), involves the replacement of exon 6 of *CFHR1* starting from the final exon of *CFH*. The third *CFH::CFHR1* fusion gene, reported by *Piras et al.* (10), involves the replacement of exons 4-6 of *CFHR1* starting from the three last exon of *CFH*. Regarding the combination of a *CFH::CFHR1* fusion gene and *CFHR* gene duplication, while *CFH::CFHR1* fusion gene and *de novo CFHR1* duplication has been previously reported (10), the simultaneous occurrence of this fusion gene with *CFHR* large duplication including the *CFHR2* and *CFHR4* gene has not been previously reported.

Here, we encountered a family with aHUS spanning four generations and identified a novel dual *CFH::CFHR1* fusion gene and *CFHR* gene duplication within one allele of the *CFH* and *CFHR* gene region. The fusion gene encompasses exons 1–22 of *CFH* and exon 6 of *CFHR1*, similar to the report by *Maga et al.* (9). However, in our case, this anomaly further included a duplication of the *CFHR3-1-4-2* genes. This extensive duplication is the first report in the world.

## 2 Case presentation of the proband

An 8-month-old girl (proband) initially presented with mild diarrhea that lasted one week. Her activity decreased and she was passing brown urine. After visiting the emergency center, she was admitted to our hospital the following morning. She was born after an uneventful 38-week pregnancy at a birth weight of 2,762 g (−0.18 SD). Her developmental milestones were normal. She appeared pale and there were signs of anemia in the conjunctiva of her eyelids. Petechiae were observed on her lower extremities, but urine volume was normal.

The laboratory results revealed the following: Hemoglobin: 6.5 g/dL, Platelet count: 7.0 × 10^3^/μL, serum creatinine (Cr): 1.27 mg/dL, lactic acid dehydrogenase (LDH): 3,932 mg/dL, total bilirubin: 2.7 mg/dL, Coombs test: negative, haptoglobin (Hp): undetectable, complement components within normal ranges (C3: 78 mg/dL, C4: 33 mg/dL), and negative anti-lipopolysaccharide (LPS)-IgM antibody. A blood smear showed the presence of fragmented red blood cells. Urinalysis indicated proteinuria (Protein/Cr ratio: 85.3 g/gCr) and hematuria. Stool cultures and occult blood tests were negative. Based on these findings, she was diagnosed with aHUS.

Treatment was initiated with a daily plasma exchange on the third day of hospitalization, resulting in the cessation of further increases in serum Cr levels and a reduction in LDH and total bilirubin; however, her platelet count remained low. By the fifth day, *Streptococcal* TMA, cobalamin C defect-HUS, and Shiga toxin-producing *Escherichia coli* infection were ruled out. ADAM metallopeptidase with thrombospondin type 1 motif 13 (ADAMTS13) activity was within the normal range.

On the sixth day, because of a positive hemolysis test, she was administered a dose of ravulizumab (Rav) at 600 mg based on her weight. Subsequently, the laboratory results showed improvement. By the thirteenth day, her platelet count had risen to 700 × 10^3^/μL, and her serum Cr level had dropped to 0.38 mg/dL. She continued to receive Rav on a monthly basis and remained disease-free for two years with no signs of recurrence.

## 3 Family history

**Figure 1:**
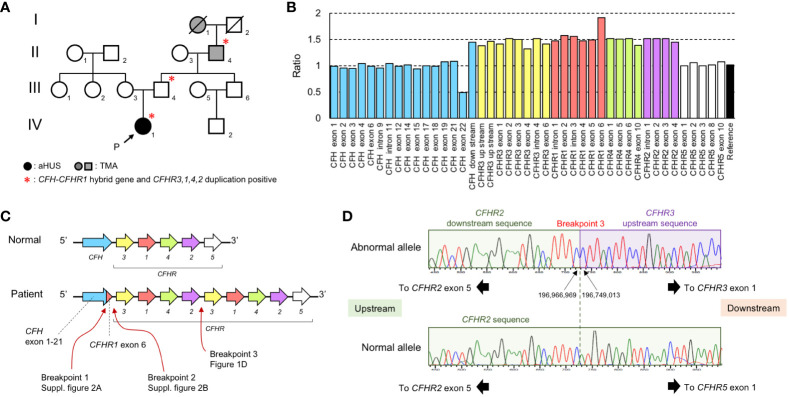
Laboratory and gene analysis of *CFH* and *CFHR*. **(A)** Family tree. There were 3 members of the family who were affected by aHUS or TMA (I-1, II-4, and IV-1) and 3 members of the family (II-4, III-4, and IV-1) carrying the *CFH::CFHR1* fusion gene and *CFHR3-1-4-2* duplication.I-1: Great grandmother II-4: Grandfather III-3: Mother III-4: Father IV-1: Proband. **(B)** The result of MLPA analysis in the patient (IV-1). MLPA analysis over the *CFH–CFHR* region shows a 1.5-fold ratio in a large region beginning with *CFH* downstream and ending *CFHR2* gene exon 4. The numbering of *CFH* exon in the graph follows the instructions for use of the SALSA^®^ MLPA^®^ Probemix P236-B1 CFH Region. **(C)** Estimation of *CFH–CFHR* region of the patient. The *CFH::CFHR1* fusion gene is caused by a gene conversion between *CFH* gene exon 23 and *CFHR1* gene exon 6. The duplication occurred from the *CFHR3* gene to the *CFHR2* gene. **(D)** Sequencing analysis of breakpoint between *CFHR2* and *CFHR3* gene (Breakpoint 3). In the normal allele, *CFHR5* exon 1 exists after the *CFHR2* downstream sequence. *CFHR3* exon 1 exists after the *CFHR2* downstream sequence in the abnormal allele. The breakpoint is located in a region 2 bp between the *CFHR2* downstream and *CFHR3* upstream region.

Her parents were nonconsanguineous and she did not have any siblings. Both of her parents were healthy and had no history of TMA; however, her paternal grandfather experienced sudden renal failure in his thirties and had been on dialysis for 40 years (**Figure 1A**). He was initially diagnosed with rapidly progressive glomerulonephritis based on a renal biopsy. Upon reevaluation of his kidney biopsy specimen, which was collected 40 years ago, it was evident that the pathology was consistent with TMA.

Five years before the proband’s birth, her paternal great grandmother suffered from anti-neutrophil cytoplasmic antibody (ANCA)-associated vasculitis in her eighties. Her renal pathology indicated necrotizing vasculitis and she was administered steroid treatment. Three months after the onset of ANCA-associated vasculitis, she developed pneumonia and hematologic TMA. The laboratory findings revealed the following: Hemoglobin: 6.5 g/dL, Platelet count: 1.7 × 10^3^/μL, Cr: 5.08 mg/dL, and LDH: 2,376 mg/dL. A blood smear showed the presence of fragmented red blood cells. Her ADAMTS13 activity was within the normal range and haptoglobin was <10%. Testing for O157-LPS yielded negative results. Unfortunately, she passed away without responding to various treatments. Although TMA was considered a possible diagnosis in her case, a definitive diagnosis was never established.

## 4 Laboratory and genetic analysis of the *CFH* and *CFHR* genes

Because TMA was identified in this family over four generations, additional tests were performed. The patient, her father, and paternal grandfather exhibited hemolytic reactions in their citric acid plasma in a hemolysis test using sheep erythrocytes (**Supplementary Figure 1**) (14). The degree of hemolysis in the citric acid plasma of the mother was similar to that of a healthy control. After the proband received Rav, the degree of hemolysis in her citric acid plasma decreased to that of the healthy control.

Genetic abnormalities in the *CFH* and *CFHR* genes were analyzed by Multiplex ligation-dependent probe amplification (MLPA). We conducted the MLPA analysis using the SALSA^®^ MLPA^®^ Probemix P236-B1 CFH Region from MRC Holland (Amsterdam, The Netherlands). The relative copy number ratio of the *CFH* exon 23 (exon 22 in the **Figure 1B**) was 0.5, the ratio of the region downstream of *CFH* to *CFHR2* exon 4 (excluding *CFHR1* exon 6) was 1.5, and the ratio of the *CFHR1* exon 6 was 2.0 (**Figure 1B**). Because this pattern followed an autosomal dominant inheritance, we hypothesized that a *CFH::CFHR1* fusion gene, in which *CFH* exon 23 was replaced by *CFHR1* exon 6, along with a *CFHR3-1-4-2* gene duplication, existed on the same allele (**Figure 1C**).

PCR and direct sequencing were done using primers specific to the *CFH* intron 22 and *CFHR1* exon 6. We confirmed the presence of a *CFH::CFHR1* fusion gene (NC_000001.11: g.196,746,919_196,748,226con196,831,605_196,832,909). The rearranged region upstream of the *CFH::CFHR1* fusion gene is presented in **Supplementary Figure 2A**. The rearranged region of downstream of the *CFH::CFHR1* fusion gene was also analyzed (**Supplementary Figure 2B**). Furthermore, we verified that the cytosine 7,347 on the 3**′** side of *CFHR2* exon 5 was continuous with the cytosine 25,827 on the 5**′** end of the *CFHR3* exon (**Figure 1D**). Based on these results, we concluded that the gene sequence of the patient consisted of a *CFH* exon 1-22*::CFHR1* exon 6 fusion gene and *CFHR3-1-4-2* gene duplication (NC_000001.11: g.196,749,013_196,966,969dup).

## 5 Discussion

In this case study, a proband and her family carried both the *CFH::CFHR1* fusion gene and a duplication of the *CFHR3-1-4-2* genes. The presence of the *CFH::CFHR1* fusion gene resulted in the replacement of the C-terminus of the FH protein with the FHR-1 protein (3, 8–10). This fusion gene encodes a hybrid FH/FHR-1 protein, in which the last SCR20 of the FH is substituted with SCR5 from FHR-1.

FH SCR20 and FHR-1 SCR5 are similar in their structural features and binding properties. Both SCR domains consist of approximately 60 amino acids and are known as a conserved domain in complement regulatory proteins (3). The C-terminal SCR5 of FHR-1 display a high degree of homology (97%) with the C-terminal SCR20 of FH, which contains a surface recognition domain (10, 15–18). As a result, FH and FHR-1 exhibit binding affinity for similar ligands, such as C3b, C3d, and cell surfaces, contributing to their roles in complement system regulation (3, 4, 15–18).

However, significant differences exist between FH SCR20 and FHR-1 SCR5, particularly in their functional implications and regulatory mechanisms. FH SCR20 binds to sialic acid, glycosaminoglycans and C3b, thus driving the recruitment of FH to cell surfaces and to the cell matrix. On the other hand, FHR-1 SCR5 strongly interacts with native C3, attracting native C3 to the proximity of the cell surface. FHR-1 is believed to exert regulatory effects through different pathways, such as modulating FH activity or interacting with other complement components (3, 4, 18, 19).

The ability of FH to bind sialic acid depends on two specific amino acids, S1191 and V1197, located in SCR20 of the FH (4, 17, 18). These residues are unique to FH and are essential for its function (18, 19). In contrast, FHR-1 contains two specific residues, L290 and A296, that are distinct from FH (4, 18, 19). FHR-1 lacks the same regulatory function of FH, which requires the N-terminus (18, 19). The *CFH::CFHR1* fusion gene preserves the FHR-1-specific C-terminal residues (18, 19). Consequently, the FH/FHR-1 hybrid protein fails to recruit FH/FHR-1 hybrid protein to cell surface and causes a loss of complement regulation on the cell surface (3, 4, 18, 19).

In addition to the *CFH::CFHR1* fusion gene, other fusion genes derived from the *CFH–CFHR* region have been identified, including *CFH::CFHR3* and *CFHR1::CFH* (3, 8, 11, 12). These genetic variations underscore the complexity of complement regulation and its implications in disorders, such as aHUS.

aHUS develops as a result of vascular endothelial cell damage caused by the activation of the complement cascade; however, not everyone with a *CFH::CFHR1* fusion gene develops aHUS. For example, the proband’s father never experienced aHUS, even though the proband’s paternal grandfather developed TMA in his thirties. Interestingly, despite the association of the *CFH::CFHR1* fusion gene with a poor clinical prognosis and a high risk of postrenal transplant recurrence, the paternal grandfather never experienced a relapse (8, 11, 12). This suggests that additional factors or triggers are likely necessary to induce aHUS in individuals with the *CFH::CFHR1* fusion gene. We have not investigated the aHUS risk haplotypes in *CFH* and *MCP* in the patient, father, and grandfather in this study. However, differences in such areas cannot be ruled out as potential contributing factors.

In addition, the patient in this case had a duplication of the *CFHR3-1-4-2* genes, which is the first reported case of such a duplication occurring in combination with a *CFH::CFHR1* fusion gene. The significance of the *CFHR3-1-4-2* gene duplication and increased FHR levels is unclear. One possibility is that FHR competitively inhibit the binding of FH to C3b and glycosaminoglycans, thereby interfering with its normal function (20, 21). Additionally, FHR-1 could also act as a complement-activating molecule (22).

The analysis conducted in the present study revealed three rearranged regions (one in the *CFH::CFHR1* fusion gene, one between the *CFH::CFHR1* fusion gene and the inserted/original *CFHR3*, and one between the inserted/original *CFHR2* and the original/inserted *CFHR3*). This indicates that one more residual ambiguous breakpoint can be technically challenging. To address this issue conclusively, it will be necessary to identify more cases with similar genetic variations.

In conclusion, this case highlights the *CFH::CFHR1* fusion gene as a potential cause of aHUS with varying severity and age of onset. The presence of a *CFHR3-1-4-2* gene duplication as observed in this case is a novel finding that provides insight into the pathogenesis of aHUS.

## Data availability statement

The original contributions presented in the study are included in the article/**Supplementary Material**, further inquiries can be directed to the corresponding author/s.

## Ethics statement

Written informed consent was obtained from the individual(s) for the publication of any potentially identifiable images or data included in this article.

## Author contributions

YT: Data curation, Investigation, Writing – original draft, Writing – review & editing. HT: Data curation, Formal Analysis, Investigation, Methodology, Writing – original draft, Writing – review & editing. TY: Conceptualization, Data curation, Methodology, Supervision, Visualization, Writing – original draft, Writing – review & editing. NS: Data curation, Investigation, Methodology, Writing – original draft, Writing – review & editing. SK: Data curation, Writing – original draft, Writing – review & editing. HF: Data curation, Writing – original draft, Writing – review & editing. YH: Formal Analysis, Investigation, Methodology, Writing – original draft, Writing – review & editing. SM: Formal Analysis, Investigation, Methodology, Project administration, Writing – original draft, Writing – review & editing. NK: Formal Analysis, Investigation, Methodology, Project administration, Writing – original draft, Writing – review & editing. NI: Conceptualization, Formal Analysis, Funding acquisition, Investigation, Methodology, Supervision, Writing – original draft, Writing – review & editing. TW: Conceptualization, Project administration, Supervision, Writing – original draft, Writing – review & editing.
