# Supplementary material for: Case report: A family of atypical hemolytic uremic syndrome involving a CFH::CFHR1 fusion gene and CFHR3-1-4-2 gene duplication
Source: Front Immunol. 2024 Mar 8;15:1360855. doi: 10.3389/fimmu.2024.1360855 (PMC10957550; doi:10.3389/fimmu.2024.1360855)
Supplement: Supplementary file 1 [file DataSheet_1.docx]

Supplementary Material

## 1. Supplementary Figures


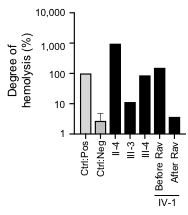


**Supplementary Figure 1.** **The degree of hemolysis of sheep erythrocytes with 20% citrate plasma (% of O72 treated normal plasma).**

Plasma from members (Ⅱ-4, Ⅲ-4, and Ⅳ-1) carrying the *CFH::CFHR1* fusion gene and *CFHR3-1-4-2* duplication lysed the sheep erythrocytes. Plasma from a member (Ⅲ-3) without gene mutation did not lyse the cells. After Rav administration, the degree of hemolysis from the proband was reduced to the same level as that of the mother and the negative control.


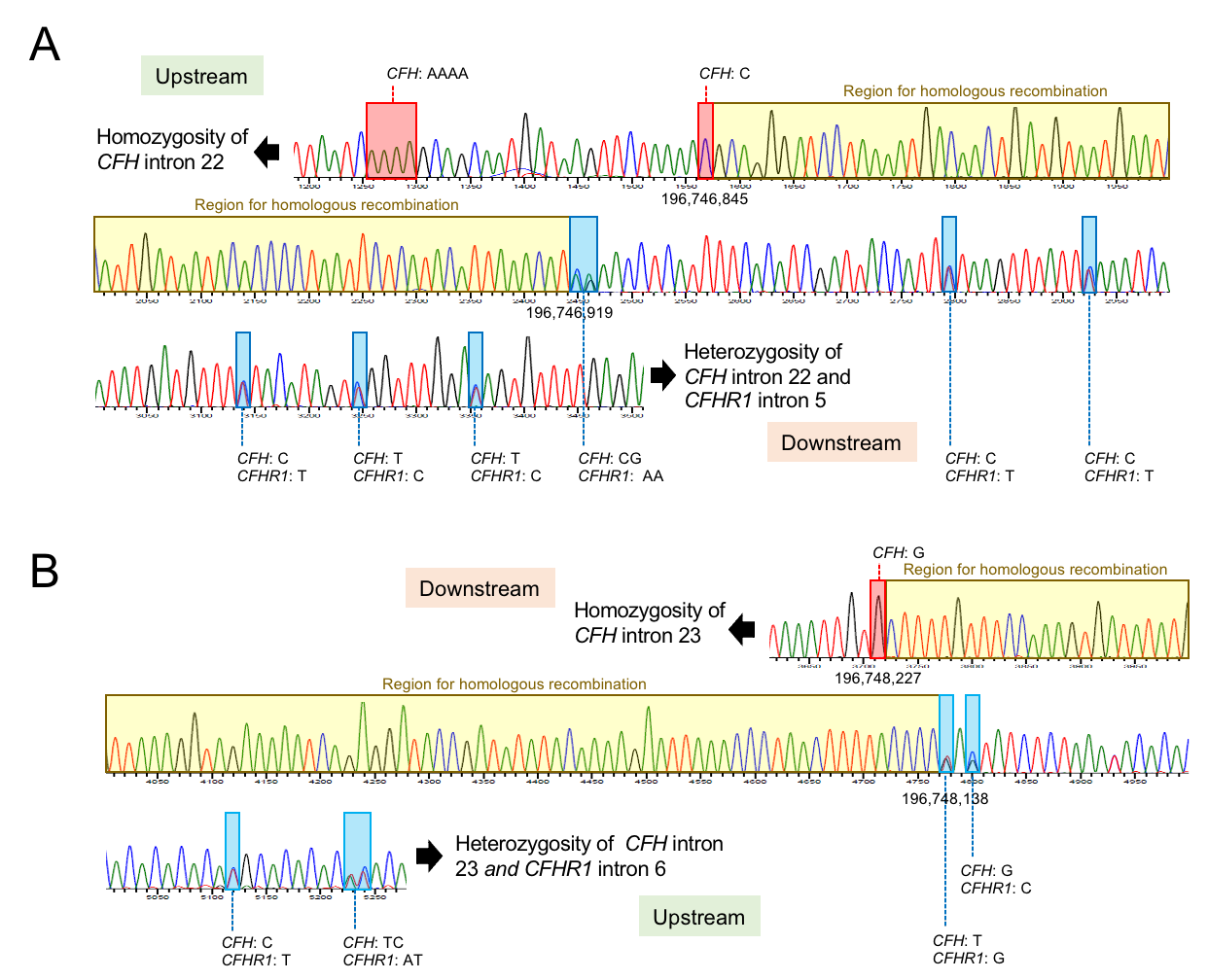


**Supplementary Figure 2. Sequencing analysis of breakpoint of *CFH::CFHR1* fusion gene**

(A) Sequencing analysis of 5′ side of *CFH::CFHR1* fusion gene (Breakpoint 1).

The rearranged region is located in a 72 bp region (yellow square) between exon 23 of *CFH* and exon 6 of *CFHR1*. The yellow square indicates the region of homologous recombination. Red squares indicate *CFH*-specific nucleotide polymorphism variants and blue squares indicate *CFHR1* or *CFH*-specific nucleotides.

(B) Sequencing analysis of 3′ side of *CFH::CFHR1* fusion gene (Breakpoint 2).

The rearranged region is located in an 87 bp region (yellow square) between exon 23 of *CFH* and exon 6 of *CFHR1*. The yellow square indicates the region of homologous recombination. Red squares indicate *CFH*-specific nucleotide polymorphism variants and blue squares indicate *CFHR1* or *CFH*-specific nucleotides.
