# Supplementary material for: Case report: A family of atypical hemolytic uremic syndrome involving a CFH::CFHR1 fusion gene and CFHR3-1-4-2 gene duplication
Source: Front Immunol. 2024 Mar 8;15:1360855. doi: 10.3389/fimmu.2024.1360855 (PMC10957550; doi:10.3389/fimmu.2024.1360855)
Supplement: Supplementary file 2 [file Presentation_1.pptx]

## Slide 1
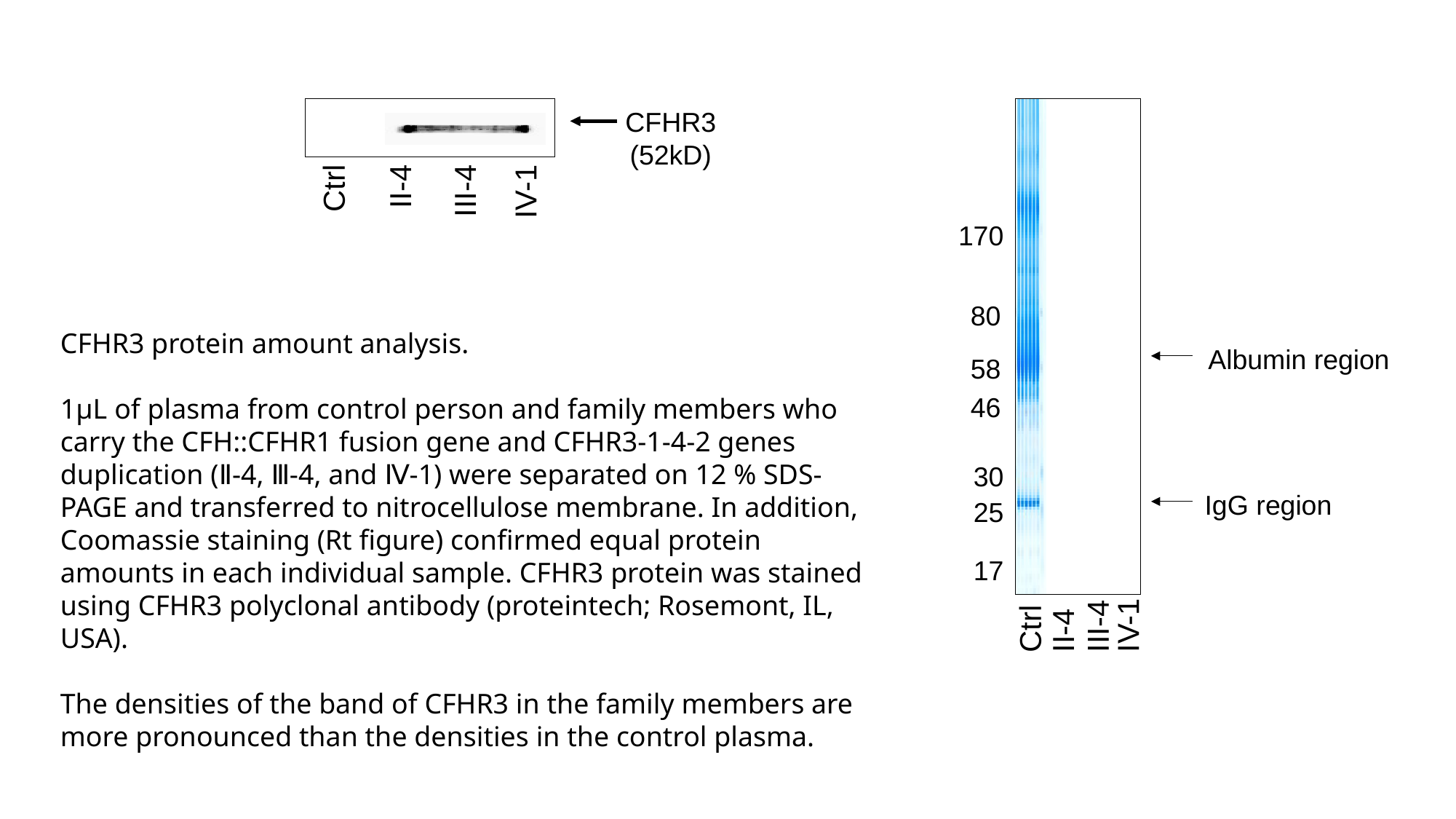

CFHR3
(52kD)
II-4
Ctrl
III-4
IV-1
170
80
CFHR3 protein amount analysis.
1μL of plasma from control person and family members who carry the CFH::CFHR1 fusion gene and CFHR3-1-4-2 genes duplication (Ⅱ-4, Ⅲ-4, and Ⅳ-1) were separated on 12 % SDS-PAGE and transferred to nitrocellulose membrane. In addition, Coomassie staining (Rt figure) confirmed equal protein amounts in each individual sample. CFHR3 protein was stained using CFHR3 polyclonal antibody (proteintech; Rosemont, IL, USA).
The densities of the band of CFHR3 in the family members are more pronounced than the densities in the control plasma.
Albumin region
58
46
30
IgG region
25
17
IV-1
III-4
Ctrl
II-4
